# Enhancement in electrical conductivity of liquid crystals by graphene metal oxide composites

**DOI:** 10.1038/s41598-023-38157-y

**Published:** 2023-07-19

**Authors:** M. Khodaee, N. Dalir, F. Feghhi, N. Ansari, M. Mohammadimasoudi, A. Goudarzi, A. F. Nasiri, M. Kolahdouz, SM. Mohseni

**Affiliations:** 1 School of Electrical and Computer Engineering, College of Engineering, University of Tehran, Tehran, 1439957131 Iran; 2 Department of Renewable Energy, Interdisciplinary Science and Technology, Tarbiat Modares University, Tehran, 14115-175 Iran; 3 Department of Physics, Alzahra University, Tehran, 19938 Iran; 4 Nano-Bio-Photonics Lab, Faculty of New Sciences and Technologies, University of Tehran, Tehran, 1439957131 Iran; 5 Department of Physics, Shahid Beheshti University, Evin, Tehran, 19839 Iran

**Keywords:** Optical properties and devices, Nanoparticles, Ferromagnetism, Structure of solids and liquids

## Abstract

Enhancing the electrical conductivity of liquid crystal (LC) circumvents challenges for application in advanced electronic components. Toward this, using additives made of different nanostructures that could result in functional LCs is suggested. In this paper, various concentrations of graphene (Gr)/metal-oxide (Fe_3_O_4_) nanocomposite (GMN) (0.0001–1 w%) were added to E7 nematic LC. We found that the role of anisotropic Gr flakes, their edges as well as surface-decorated-metal-oxide-additives have significant impact on electrical properties of E7. A range of appropriate additives of such a nanocomposite enhances the electrical conductivity of LCs. This effect can be traced through the decrease in the formation of GMN aggregates in the E7 and increase in the electrostatic field at the edges of the Gr sheets. Moreover, the presence of metal-oxide nanoclusters due to the presence of oxygen vacancies and defects facilitates the construction of conductive network for improving the charge transfer pathways and contributes to a stronger interaction of the Gr surface with charged species. These factors can provide Gr layers as dipole moments and lead to signal propagation in the dielectric medium. Our finding conveys a pathway toward significant enhancement of electrical conductivity in the LC family which can be useful for functional applications.

## Introduction

Doping liquid crystals (LCs) with nanomaterials addresses an important strategy for tuning their properties^1^. Doped LCs exhibit significantly improved properties compared to their undoped ones, while they also achieve long-term stability for industrial applications^2–5^. It has been shown that low loadings of various nanomaterials with zero-, one-, and two-dimensional structures dispersed in LCs media can significantly affect their physical properties, especially space-charge distribution^6–11^. For instance, electro-optical response as well as the electrical behavior of LCs can be influenced by such doping^12,13^. In this regard, two major factors of nanomaterials, i.e. their surface-to-volume ratio, as well as their interactions between active agents and ions of LCs can make them promising candidates for demanding applications^14^.

Improvement of electrical conductivity of LCs due to doping can also influence their properties for more functional applications^15^. To date, this has been achieved through the inclusion of high concentrations of nanomaterials in LCs which can lead to the amplification of ionic conductivity^11^ and degradation of the electro-optical response due to aggregate formation^16^. Moreover, in the cases of chain and network formation, the enhancement in DC conductivity can disturb the LC properties. Studies have shown this effect for LCs doped by nanoparticles made from carbon nanotubes, metals, and polymeric inclusions^6,17–19^. Alternatively, better exploitation of liquid crystalline behavior for demanding applications needs an understanding of ionic phenomena in LCs doped with nanomaterials^20,21^ and identification of processes that lead to the purification of LCs with optimal values of nanomaterials^14,15,22,23^. Considering the attempts leading to optimization of LC properties in some fields by functionalizing nanoparticles^11,24,25^, purification along with conductivity improvement requires using low concentrations of nanomaterials in LCs. Hence, nanomaterials need to be surface-treated differently^26^ for tuning their effective role within LCs.

Graphene (Gr) is one of the well-known 2D nanomaterials that can manipulate the electrical properties of LCs^27–37^. The effectiveness of Gr layers in different phases of LCs often leads to two outcomes: (I) When Gr sheets mix with LC, their electrostatic field and screening effect leads to the suppression of ionic behaviors such as reduction in ionic density, diffusivity, conductivity, and relaxation frequency through the ion trapping/charge annihilation process^22^; (II) The interaction between the honeycomb pattern of Gr and the benzene rings of LC molecules causes the LC directors to planar stabilize on the surface of the Gr layers and results in the improvement of dielectric anisotropy^38^.

However, in both cases, the function of Gr is limited to serving as alignment layers for molecules of host LCs. Moreover, considering that strong van der Waals interactions between Gr layers tend to cause Gr re-stacking together with the oxidation effects during the synthesis process, these problems lead to the loss or even deprivation of the diffusion rate and excellent specific surface area of Gr, limiting its applications in various fields^39–41^.

One of the most efficient solutions to overcome these problems is to functionalize Gr sheets with oxygen-containing groups such as transition metal-oxides with excellent oxygen evolution reactions (OER)^42^. This not only enhances the adsorption process but also facilitates the construction of a 3D porous conductive network for improving the charge transfer pathways and electrical properties^43,44^. In addition, the Gr surface provides nucleation sites for the in-situ growth of the metallic nanoparticles between their successive layer formation that prevents their agglomeration^45,46^. Such metal-oxide formation decorated at the surface of Gr layers includes vacancies and defects that play as active sites adjacent to LCs for improving conductivity and inducing functionality.

It has also demonstrated nanomaterials of Gr and metal-oxide agents are considered to be potential candidates for charge trapping and their electric field-controlled effects^47^. Family of various metals such as Fe, Cu, and Ag can be suggested to provide active sites adjacent to Gr plates. They are thus suitable for deployment as doping agents in LCs to control LC response against the electric field. Interestingly, migration of oxygen vacancies and metal cations via the electric field in such materials influences chemical activity and conductivity of the product^48,49^.

To achieve LCs with controlled conductivity through doping agents, in this paper, we used air-stable multi-layer Gr (MLG) hosting metal-oxide Fe_3_O_4_-nanoclusters as an additive to E7 nematic LC. This Gr/metal-oxide-nanocomposite (GMN) is achieved through the electrochemical exfoliation of graphite^50–52^, which produces a scalable Gr product and the simultaneous deposition of Fe_3_O_4_ nanoclusters on the surface of Gr layers in a suitable solution^53^. Different concentrations of this hybrid within the E7 are investigated to realize the dual purpose of prevention of aggregation formation in LC and improvement of its conductivity, using polarizing optical microscopy (POM) and also the powerful impedance spectroscopy tool.

## Results and discussion

**Figure 1:**
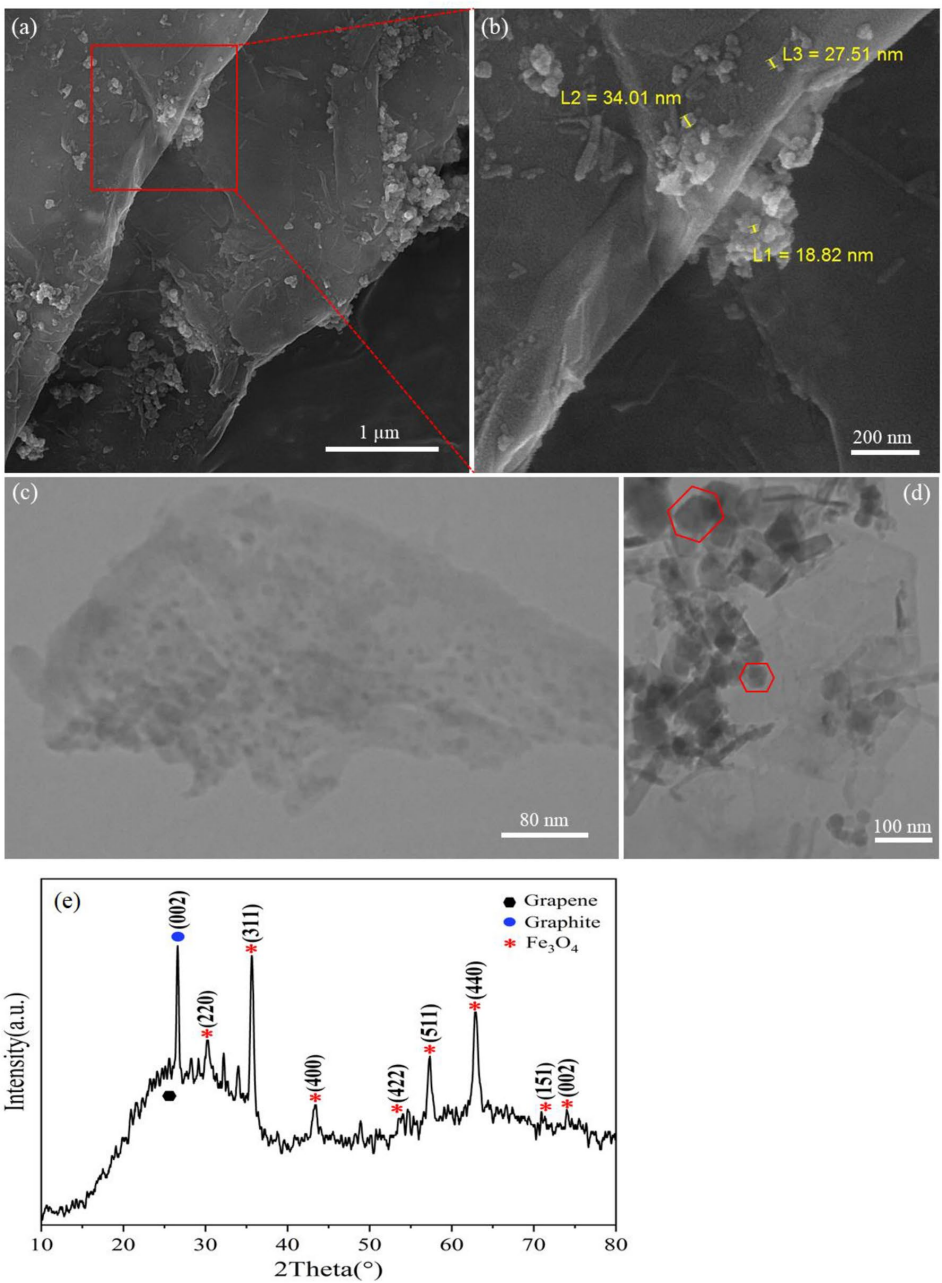
(**a**,**b**) FESEM images of GMN and different size of Fe_3_O_4_ nanoclusters especially in the edges of the Gr layers and (**c**,**d**) TEM images of a micron boundary size of Gr sheet and different geometry of Fe_3_O_4_ samples indicating well distribution of Fe_3_O_4_ nanoclusters on the Gr layers. (**e**) XRD pattern of GMN, showing well exfoliation of graphite and formation of the Gr layers.

Figure 1 shows the results of the GMN characterization. The FESEM and TEM analysis (Fig. 1a–c) validate the formation of micron boundary size of Gr multi-layered structure and Fe_3_O_4_ nanoclusters distributed randomly on its layers. In Fig. 1b, the closer we get to the edges of Gr layer, the more aggregations of nanoclusters are formed. Although, the dimensions of some of them are below 35 nm. Figure 1d shows another TEM view of nanoclusters near the edge of the Gr layer. According to this analysis, there are hexagonal structures, some of which are stacked or rolled up, which are related mainly to Fe_3_O_4_ nanocrystals. Furthermore, the examination of the XRD pattern of this nanocomposite confirms the presence of Fe_3_O_4_ peaks at defined 2θ positions. Also, the presence of these peaks on a non-smooth line may indicate the formation of some of the Fe_3_O_4_ structures in the amorphous phases. This data also shows the typical crystalline structure of graphite in the material. In fact, the XRD pattern of graphite has a sharp and tight peak at 26° which corresponds to the diffraction line C (002)^54^. However, through the exfoliation process, this high-intensity characteristic has degraded to some extent, and instead a broad mound corresponding to the graphene is added.

**Figure 2:**
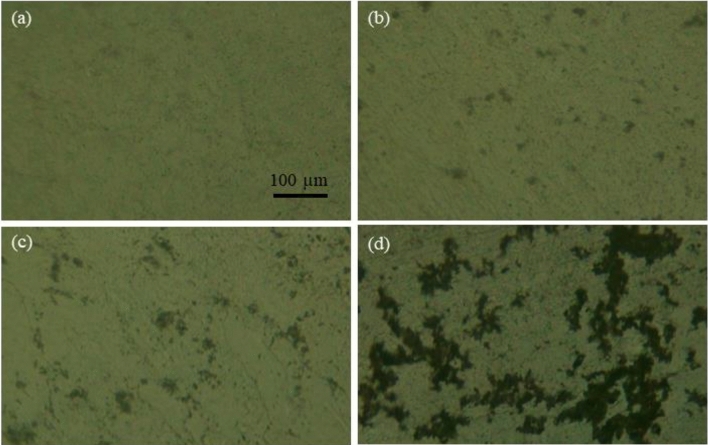
The images of texture changes in (**a**) 0.0001 w%, (**b**) 0.001 w%, (**c**) 0.01 w%, (**d**) 1 w% GMN-doped samples observed under POM. The cells are placed between parallel polarizer and analyzer.

The POM images of texture changes for dispersions of GMN in E7 at different concentrations (0.0001–1 w%) have shown in Fig. 2. The 0.0001 w% sample (Fig. 2a) has a relatively uniform texture, indicating a homogeneous distribution of the nanocomposite within the LC. In fact, in this case, the concentration of nanocomposite is low enough to minimize aggregation formation within the LC. In the 0.001 w% sample (Fig. 2b), aggregates with maximum dimensions of 20 μm appear apart from each other. In the 0.01 w% sample, these aggregates connected in places, and finally, in Fig. 2d, the sample was dominated by separately large aggregations of the GMN. Although there is no trace of the chain formation of aggregates up to the concentration of 1 w% GMN dopant in the E7, these aggregates affect the electrical properties of the samples, especially for dopant concentrations of (0.01–1 w%), which will be discussed later.

**Figure 3:**
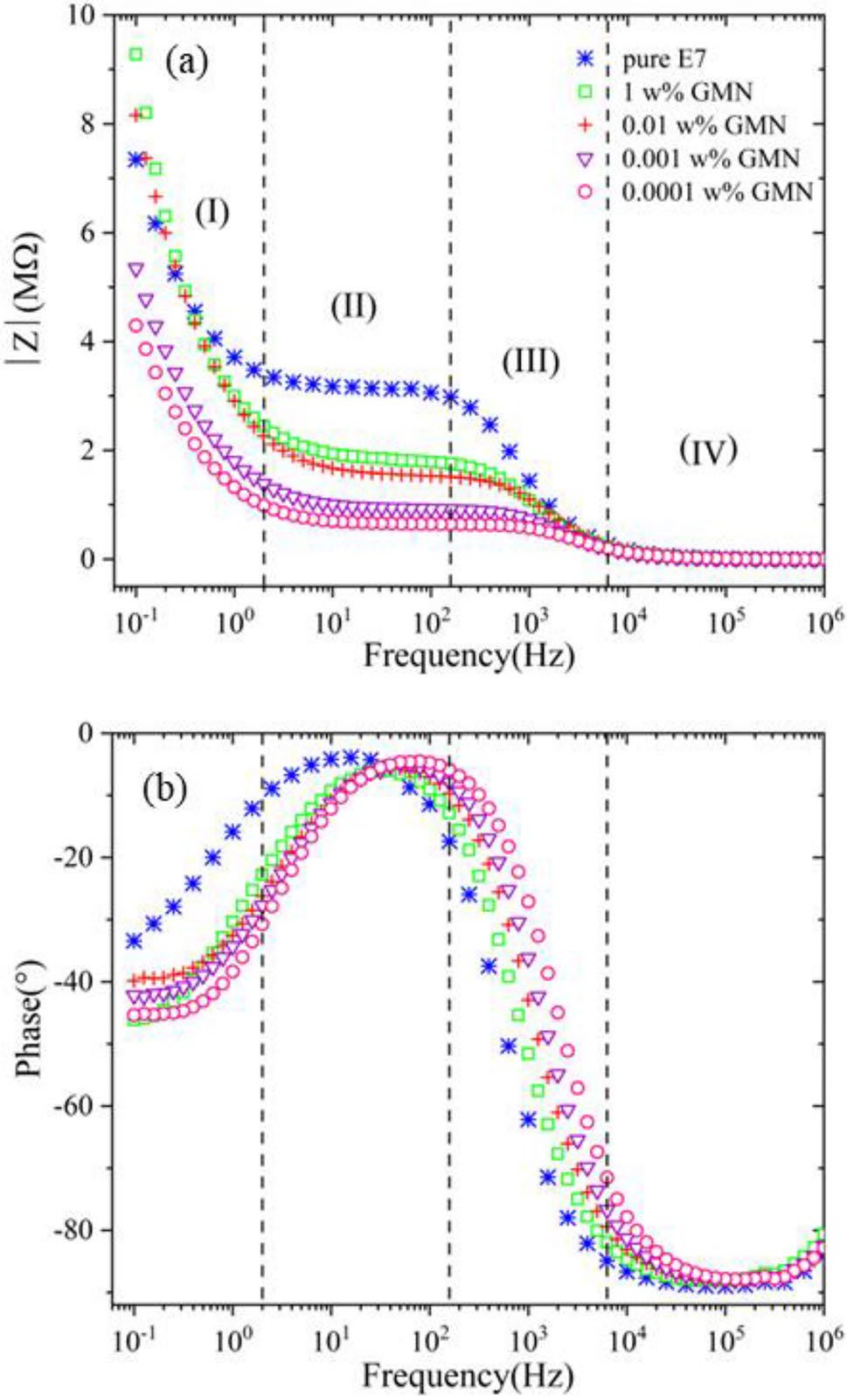
(**a**) Impedance magnitude and (**b**) phase angle vs. frequency for pure and GMN-doped E7.

Impedance spectroscopy is an efficient method for tracking the behavior of mobile ions in the bulk and interfacial regions of fluids. Experimental data for the magnitude $|Z|=\sqrt{{Z}_{r}^{2}+{Z}_{i}^{2}}$ and phase angle Φ = arctan ${(Z}_{i}/{Z}_{r})$ of complex impedance, in which Z_r_ denotes the active component, and Z_i_ denotes the reactive component, are depicted in Fig. 3. The process of changing the impedance value for pure E7, as depicted with blue symbols in Fig. 3a, can be divided into four parts in the overall frequency range. As the frequency increases from 100 mHz to 2 Hz (part I), the magnitude of impedance has a decreasing trend and then this slope flattens out and gets a plateau of a relatively-constant value over a broad frequency range (2–158.5 Hz) (part II). For frequencies above 158.5 Hz up to nearly 6.3 kHz (part III), the second linearly decreasing trend occurs. Finally, for frequencies more than 6.3 kHz (part IV), the magnitude of the impedance reaches a constant value. The phase angle for frequencies more than 6.3 kHz remains within the extent of − 85° to − 89° and as the frequency decreases, the phase response reaches a maximum with the value of − 3.89°, which corresponds to the saddle point at the frequency of about 15.85 Hz, and then it decreases until the reach to the frequency of 100 mHz. In other words, from 100 mHz to 15.85 Hz, molecules present at the interface interact with the electric field, but in the range 15.85 Hz to 1 MHz, the LC dipole moment begins to interact with the electric field^13,55^.

As shown in Fig. 3, the addition of GMN with different concentrations (0.0001–1 w%) to the E7 leads to a shift in the impedance data to higher frequencies. Of course, the lower the dopant concentration, the more the frequency shift. More details are as follows: The impedance value and phase response, for all of the doped samples in part IV, tend to be constant nearly to the pure E7 response, which results in the conclusion that in this part, the reactive component of the impedance is dominant and we are facing primarily capacitive behavior. In the first three parts with decreasing frequency, the impedance values of the doped samples follow the general trend of pure E7. However, their impedance values are lower compared to E7, and the smaller the amount of GMN dopant, the more the drop in impedance values. This general principle continues in the first part down to a frequency of 0.25 Hz, and then for the lower frequencies is broken by the 1 w% and 0.01 w% samples whose impedance values exceed that assumed by pure E7. In Table 1S (SI), these changes were analyzed quantitatively. As seen in Fig. 3a, the behavior of doped samples is divided into two categories due to the similarity of the responses in impedance magnitude. Those samples inside the range of (0.01–1 w%) are in the first category and those within the range of (0.0001–0.001 w%) are in the second one. The main difference in appearance between these two categories is the size of aggregations which will be discussed later. One can derive the behavior of a capacitor with a residual leakage current from the impedance response of the diagrams in Fig. 3.

**Figure 4:**
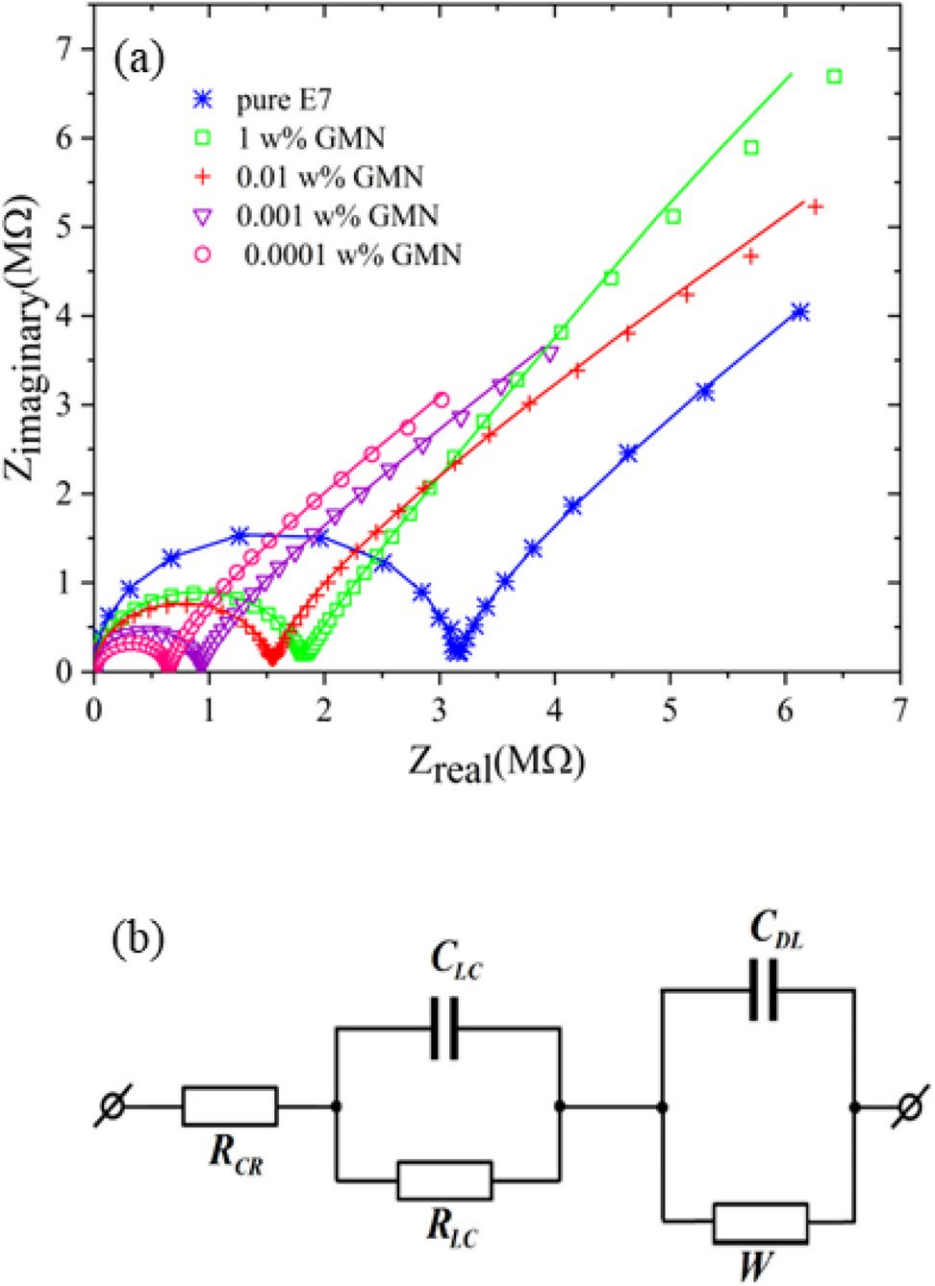
(**a**) The Cole–Cole plots and (**b**) equivalent electrical circuit model for pure and GMN-doped E7. The fitted lines are shown by solid lines in part (**a**).

To get a better idea of the electrical behavior of the samples, the Cole–Cole plots are also included in Fig. 4a. The Cole–Cole plot of pure E7 appears in a semicircle and a Warburg impedance, and this form of reaction has been confirmed by all of the GMN-doped samples. This semicircle represents the mid-range frequency response of the system, and its diameter indicates the bulk resistance of the LC, which is reduced by the addition of the GMN dopant. Noteworthy is the direct relationship between the dopant concentration and the magnitude of its bulk resistance. The lower the dopant concentration, the more the bulk resistance decreases.

A quantitative review of the above-mentioned topics is provided by presenting the electrical equivalent circuit (EEC) model in Fig. 4b. Here, the EEC model can be defined as the series connection of the following three parts: the part related to the resistance of the electrodes and connectors R_CR_, the part related to the high frequencies (100 Hz–100 kHz) caused by the reaction of LC molecules to the electric field and characterized by the parallel connection of R_LC_, the active component and C_LC_, the reactive component of the bulk of LC^17,56,57^, and the part associated with low frequencies (100 mHz–100 Hz) which considered the reaction of spaced charges in the vicinity of the electrodes and consists of the parallel connection of C_DL_, the double layer capacitance and W, finite diffusion Warburg element^57–59^. The Warburg element itself consists of two parts: W_sr_, Warburg coefficient in bulk and W_sc_, Warburg coefficient in double layer^11^. More details are available in the SI.

The solid lines in Fig. 4a depict the good-fitting results of retrieving the cole–cole plot for each sample using this EEC model, and Table 1 displays the values obtained for each component. The obtained results are as follows: The resistance of connectors and electrodes R_CR_ is not affected by doping of GMN (R_CR_ = 0.2 ± 0.04 kΩ). Furthermore, the reactive component of the bulk, C_LC_, which is related to the permittivity of the LC, remains constant upon dopant addition (C_LC_ = 110.1 ± 14.1 pF). Albeit a small deviation is expected from slightly different cell gaps. The data obtained from C_LC_ were also used to calculate the real, ε_r_, and imaginary, ε_i_, part of the complex dielectric permittivity, ε = ε_r_ − iε_i_, and its magnitude, Δε, for pure and doped E7 samples, which generally indicates that the values obtained for each dielectric parameter of doped samples are higher than the values obtained for pure one. Considering that these parameters were obtained at frequencies at which ionic contaminants and spaced charges cannot follow the polarization of the applied electric field, these results are reasonable.

**Table 1:** Nominal parameters for the elements of the EEC model as obtained by fitting the EEC response to the experimental data.

Sample	R_CR_ (kΩ)	R_LC_ (MΩ/cm^2^)	C_LC_ (pF)	C_DL_ (nF)	W_sr_ (MΩ·s^−1/2^)	W_sc_ (Ω·s^−1/2^)	ε_r_	ε_i_	Δε
Pure E7	0.163	3.12	96.85	75.8	31.23	45.36	4.2	3.49	5.46
1% GMN	0.241	1.78	112	20.5	39.28	18.88	4.99	4.97	7.04
0.01% GMN	0.188	1.53	96	35.28	39.58	49.69	4.15	4.47	6.10
0.001% GMN	0.169	0.92	124.2	55.33	26.12	40.77	5.60	6.17	8.33
0.0001% GMN	0.160	0.646	121.5	82.37	29.60	69	5.38	5.45	7.65

In addition, in the low-frequency part, due to the dispersion in the values of the double-layer capacitor, a clear relationship between it and the dopant concentration cannot be obtained. Of course, C_DL_ is three orders of magnitude larger than the bulk capacitance and dominates the impedance spectra at sufficiently low frequencies.

A strong effect of GMN doping is found for the bulk resistance, R_LC_, which is caused by residual impurities in the LC. It decreases from 3.12 MΩ/cm^2^ for pure E7 to 0.646 MΩ/cm^2^ for 0.0001 w% sample. This decrease in bulk resistance of the samples can be equated with the increase in the relevant electrical conductivity^60^. The inverse trend of increasing this electrical conductivity with decreasing dopant concentration indicates that ionic impurities have no significant contribution to this increasing trend. Furthermore, considering the planar alignment of the Gr sheets in accordance with the E7 nematic director, to reduce the elastic distortion of the nematic matrix, these sheets act as insulators for the propagation of the applied electric field^22^. Therefore, improving conductivity using a low concentration of GMN dopant should be sought after.

(i) LCs are insulators and their conductivity is due to the presence of ionic impurities nominated as ionic conductivity^7^. Gr sheets are potential source of ionic impurities and near their edges electrostatic field is strong. This is more significant when Gr sheets are corrugated or they have small lateral sizes^61^. Hence, they can trap ionic impurities in dielectric media and reduce the ionic conductivity. Meanwhile, the effects related to metal-oxide nanoclusters between Gr layers cannot be ignored. The presence of Fe_3_O_4_ nanoclusters, in addition to enriching the surface of Gr layers with metal ions, also increases its interaction in the LC medium. At sufficiently low frequencies, different redistributions of oxygen vacancies and defects in these nanoclusters in response to the applied electric field lead to the formation of nano-conductive areas on the surface of Gr layers which facilitate the charge transfer^47,62,63^. These effects are particularly pronounced at the edges of Gr layers, which tend to accumulate more charged species. These factors can make Gr layers act as dipole moments, resulting in the creation of dipolar fields and signal propagation in the direction perpendicular to the cell. Moreover, the smaller the aggregations, the more effective areas are available for the Gr layers to absorb the space charges thus leading to the enhancement of conductivity. This is important to note that GMN dopant can act as a source of ions and upon dispersing them within the LC, some fraction of these ions can be released into the bulk. However, the used E7 LC initially contains a considerable amount of ionic impurities, and its electrical conductivity is in the range of 10^−7^ S/m. Moreover, investigation of the process of changes in bulk resistance (or related electrical conductivity) value in all samples especially those with the lowest amount of dopant concentration, suggests the dominant behavior of ion trapping by dopants^21^.(ii) Gr is able to anchor active components to its surface. Gr’s large area acts as an alignment layer that exerts another anchoring successively and induces a short-range orientational order within the LC molecules. The origin of this effect lies in electron π–π stacking and is achieved through the matching of the benzene rings of LC molecules to the Gr honeycomb pattern. The interesting effect of this anchoring is a substantial charge transfer from the LC molecule to the honeycomb pattern of the carbon atoms in the Gr anisotropic domain and a reduction in suspension energy. Also, the presence of Fe_3_O_4_ nanoclusters, through increasing the porosity of the MLG, contributes to this function. This phenomenon can be considered a factor in increasing the conductivity in the LC. Contrarily, this orientational order is locally disturbed by increasing the concentration of GMN dopant in the LC and aggregates formation. This phenomenon is confirmed by the Cole–Cole diagrams in Fig. 4a.

**Figure 5:**
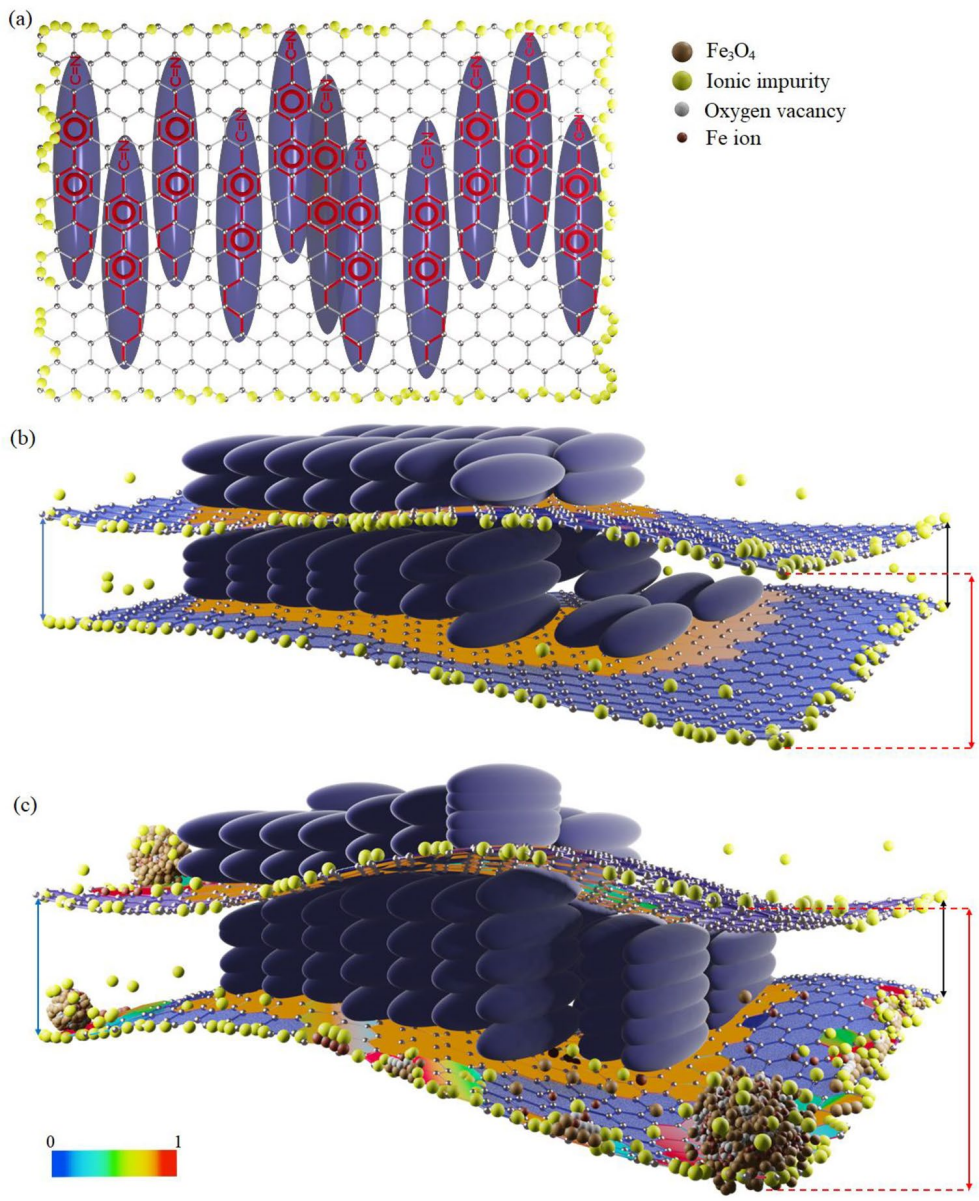
The extended plane composed of repeating unit cells in the shape of a hexagon with carbon atoms at its corners represents the Gr sheet and the blue ellipsoids represent the LC molecules. (**a**) Matching of the benzene rings of LC molecules to the Gr honeycomb pattern and ion trapping effect at the edges of the Gr sheet. (**b**) Corrugated multi-layer structure of Gr exerts another anchoring to the LC components and induces short-range orientational order within LC molecules, and (**c**) the presence of metal-oxide nanoclusters, defects, and oxygen vacancies between Gr successive layers formation prevent their agglomeration. For clarity, the upper-right corner of the figure shows the defect, charged species, and metal-oxides with colored spheres which are larger than the dimensions used in the Gr sheet. The charge transfer has been indicated by a color map on the surface of Gr, with its density specified with different colors of a color box located at the lower-left corner of the figure. The associated numbers of the color box are representatives of the minimum and maximum of this phenomenon, respectively. In part (**b**), charge transfer is due to the presence of LC molecules on the surface of Gr. Furthermore, in part (**c**), the addition of metal-oxide nanoclusters and the defects through them leads to the formation of conductive pathways and charge transfer to the Gr sheets at sufficiently low frequencies of the applied electric field. This phenomenon is pronounced at the edges of Gr and turns it into an electric dipole, indicated by the different colors of the color box around the nanoclusters.

For more clarity, we address mentioned phenomena by the schematics prepared in Fig. 5. Figure 5a shows the matching of LC benzene rings with the honeycomb pattern of Gr. In Fig. 5b, we can also see the corrugated layer of Gr acts as an alignment layer for LC molecules. In both of Fig. 5a,b, the edges of Gr, due to the electrostatic field, purify the LC from the charged species that were added to the LC due to the passage of time or through different manufacturing processes. But the interesting effect happened in Fig. 5c, which shows metal-oxide nanoclusters that affected the van der Waals attraction between the Gr layers to some extent. This effect has provided the multi-layered structure of Gr with more porosity for anchoring the LC components and enhances its molecules' orientational order. Furthermore, at the points where nanoclusters present and especially at the edges of Gr, there are defects that create more attraction for charged species (different colored spheres have been used to indicate defects and charged species) of the LC and, at sufficiently low frequencies of the applied electric field, form networks for charge transfer to Gr sheets. The set of these phenomena leads to a substantial charge transfer to the Gr surface, especially on its edges and supports its layers as electric dipoles in the dielectric medium. We prepared a color map showing the charge distribution on the Gr sheets in Fig. 5b,c. The hypothetical values of each color have been identified in the lower-left corner of Fig. 5. In Fig. 5b, the boundaries of this color pattern are limited around the LC molecules on the surface of Gr. But in Fig. 5c, the addition of nanoclusters and the processes attributed to them in the presence of the applied electric field has increased the intensity of this color map, especially at the edges of Gr sheet.

Here, in the first category, (0.01–1 w%) samples, based on the results of POM analysis, it has been observed that as the concentration of GMN increases to 0.01%, aggregation occurs between these GMN dopants. As a result, the amount of available surface decreases and the adsorption of ionic impurities onto GMN dopants decreases. This leads to a decrease in electrical conductivity compared to lower concentrations in which smaller aggregation occurred between GMN dopants. In the second category, (0.0001–0.001 w%) samples, it is also possible to highlight the profound effect of separate small dipoles of multi-layered Gr on the reaction to the electric field and even the 0.0001 w% sample may be introduced as the optimal concentration among the others, in terms of the effect on the electrical parameters of the suspension.

## Conclusion

In summary, we demonstrated aggregate formation by adding different concentrations of GMN, (0.0001–1 w%) to E7 nematic LC using POM. Also, using the powerful impedance spectroscopy tool, we studied the electrical parameters of GMN-doped samples and have shown that the electrical conductivity of these samples improved in the range of doping concentrations compared to the pure E7 one. This effect is pronounced especially in the 0.0001 w% sample. We attributed it to the reduction in aggregate formation and also the presence of metal-oxide nanoclusters between Gr layers. These factors provide the Gr surface with a large area for interaction and make it active through substantial charge transfer. For future works, considering the magnetic properties of Fe_3_O_4_ nanoclusters, it is suggested to investigate the electrical properties of the sample with optimum concentration of GMN dopant in the presence of a magnetic field. It is also possible to check the memory effects of such a fluid by sweeping the electric field at a higher amplitude.

## Methods

In this study, the nanocomposite of porous expanded multi-layer Gr structure with Fe_3_O_4_ nanoclusters entrapped between the Gr layers, Gr/metal-oxide-nanocomposite (GMN), was utilized as the dopant to the commonly known, E7 nematic LC with nematic to isotropic temperature 58 °C.

### GMN fabrication

The GMN production process is based on a simultaneous electrochemical exfoliation/deposition procedure by using a two-electrode system with nickel as the cathode electrode and graphite foil as the anode electrode. FeSO_4_·7H_2_O powder (5.56 g) and NaOH tablets (2.8 g) dissolved in distilled (DI) water (200 ml), were used to prepare the electrolyte. Controlling the electrode potentials results in the expansion, exfoliation of graphite, and deposition of iron oxide nanoclusters between Gr layers. After 3 h of DC bias (10 V), the achieved product is collected by a magnet from the solution, washed with DI water, and dried at 100 °C.

### Suspension preparation

To prepare suspensions with different concentrations of nanocomposite in E7, 2 mg of GMN powder was dispersed in 60 ml of methanol and sonicated in the ultrasonic bath for 1 h. Then we added specific amounts of this dispersion to the E7 at the isotropic temperature. The resulting compounds were further sonicated in the bath at 75 °C for 2 h until the GMN had dispersed in the E7. To evaporate the remaining methanol, we put the resulting suspensions in the oven at 135 °C for 2 h. The final suspensions obtain with four concentrations of 1 w%, 0.01 w%, 0.001 w%, and 0.0001 w% of GMN in E7.

### Sample preparation

For impedance characterization, freshly prepared suspensions were filled at 70 °C into PVA-coated test cells with a 38 µm cell gap, 1 cm^2^ electrode area, and antiparallel rubbing for planar alignment with the capillary method. For comparison, another cell with the same structural properties is filled with pure E7. For convenience, the samples with different concentrations of GMN will be abbreviated as 1 w% sample, 0.01 w% sample, 0.001 w% sample, and 0.0001 w% sample in the text.

### Characterization

The imaging of the nanocomposite was obtained by using field emission scanning electron microscope (FESEM) and tunneling electron microscopy (TEM). The crystallinity of nanocomposite confirms using the X-ray diffraction (XRD) patterns. For all of the prepared samples impedance spectroscopy and conductivity measurements were recorded using a sine-like voltage with an amplitude of 0.3 V (RMS) and a frequency in the range of (100 mHz–1 MHz) applied to cells in out of plane manner utilizing NOVA model 2.1.5 (Made in the Netherlands) connected to a personal computer. The texture of the GMN-doped samples was observed using a POM when the cells were between parallel polarizer and analyzer. All measurements were done in the nematic phase at room temperature (RT).

## Supplementary Information

Supplementary Information.

## Data Availability

The data supporting the findings of this study are available from the corresponding author upon request.
