# Supplementary material for: Enhancement in electrical conductivity of liquid crystals by graphene metal oxide composites
Source: Sci Rep. 2023 Jul 19;13:11688. doi: 10.1038/s41598-023-38157-y (PMC10356762; doi:10.1038/s41598-023-38157-y)
Supplement: Supplementary file 1 — Supplementary Information. [file 41598_2023_38157_MOESM1_ESM.docx]

**Supplementary Information (SI)**

**Enhancement in electrical conductivity of liquid crystals by graphene metal oxide composites**

**M. Khodaee^1^, N. Dalir^2^, F. Fegghi^3^, N. Ansari^3^, M. Mohammadimasoudi^4^, A. Goudarzi^4^, A. F. Nasiri^1^**

**M. Kolahdouz^1*^, S. M. Mohseni^5**^**

^1^ School of Electrical and Computer Engineering, College of Engineering, University of Tehran, Tehran1439957131, Iran

^2^ Department of Renewable Energy, Interdisciplinary Science and Technology, Tarbiat Modares University, Tehran14115-175, Iran

^3^ Department of Physics, Alzahra University, Tehran 19938, Iran

^4^ Nano-bio-photonics Lab, Faculty of New Sciences and Technologies, University of Tehran, Tehran1439957131, Iran

^5^ Department of Physics, Shahid Beheshti University, Evin, Tehran19839, Iran

Table 1S. The process of quantitative changes related to impedance value

| sample | Slope of impedance value in the part (I), (MΩ/Hz)  Measured in the frequency range of (0.1Hz-1Hz) | quasi-constant impedance value in part (II),  (MΩ) | Slope of impedance value in part (III), (MΩ/kHz)  Measured in the frequency range of (1kHz-6.3kHz) |
| --- | --- | --- | --- |
| Pure E7 | -4.03 | 3.16 | -0.23 |
| 1 w% sample | -7.077 | 1.85 | -0.17 |
| 0.01 w% sample | -5.83 | 1.56 | -0.17 |
| 0.001 w% sample | -3.937 | 0.92 | -0.1 |
| 0.0001 w% sample | -3.301 | 0.64 | -0.071 |

The changes in slopes and impedance values in the first three parts have shown in table (1S). In part III, the decrease in the slopes of 1 w%, 0.01 w%, 0.001 w%, and 0.0001 w% samples compared to pure E7 are 35.29%, 35.29%, 56.52%, and 69.56%, respectively. In part I, we are facing a different trend. The response slopes for 1 w% and 0.01 w% samples increase compared to the pure E7. The increases are 75.6% and 44.66% for 1 w% and 0.01 w% samples, respectively. Therefore, at the frequency of 0.25 Hz, the impedance values of these two samples exceed the value of pure E7 response, and at the final frequency of 100mHz, the impedance values for 1 w% and 0.01 w% samples and pure E7 are 9.2 MΩ, 8.15 MΩ, and 7.34 MΩ, respectively. We have also examined suspension with a higher concentration of GMN dopant, in which both the frequency of overtaking of the response and the magnitude of the low-frequency impedance compared to pure E7 increase significantly above these values. Albeit, we did not find it necessary to present this result. At the opposite point, the response of 0.001 w% and 0.0001 w% samples are different, and their slopes are reduced compared to the pure E7 sample. The values of these slope reductions for 0.001 w% and 0.0001 w% samples are equal to 2.3% and 18.09%, and at the frequency of 100 mHz, the impedance values for them are 5.34 MΩ and 4.29 MΩ, respectively.

**Warburg Impedance**

The generalized finite-length Warburg impedance $Z_{w}$ is expressed by the following expression[1]:

$$Z_{w}=\frac{W_{sr}}{\sqrt{iw}}(1-i)\tanh(W_{_{sc}}\sqrt{iw})$$

With the two parameters $W_{sr}$and $W_{sc}$as follow:

$$\begin{matrix} W_{sr}=\frac{RTN_{A}}{F^{2}An_{s}\sqrt{2D}} \\ W_{sc}=\frac{\delta_{n}}{\sqrt{D}} \end{matrix}$$

where R is the gas constant, T absolute temperature, N_A_ Avogadro’s constant, F the Faraday constant, A the electrode area of the test cell, n_s_ the concentration of mobile ions on the surface, D the effective diffusion coefficient of mobile ions, δ_n_ the thickness of the Nernst diffusion layer, W_sr_ Warburg coefficient in bulk and W_sc_ Warburg coefficient in double layer.

**References**

[1] G. J. Sprokel, “Resistivity, permittivity and the electrode space charge of nematic liquid crystals,” *Mol. Cryst. Liq. Cryst.*, vol. 22, no. 3–4, pp. 249–260, 1973.
